# Impact of strain, pressure, and electron correlation on magnetism and crystal structure of Mn_2_GaC from first-principles

**DOI:** 10.1038/s41598-020-68377-5

**Published:** 2020-07-09

**Authors:** Martin Dahlqvist, Johanna Rosen

**Affiliations:** Thin Film Physics, Department of Physics, Chemistry and Biology (IFM), Linköping University, 581 83 Linköping, Sweden

**Keywords:** Ferromagnetism, Electronic structure, Magnetic properties and materials, Metals and alloys, Coarse-grained models

## Abstract

The atomically laminated Mn_2_GaC has previously been synthesized as a heteroepitaxial thin film and found to be magnetic with structural changes linked to the magnetic anisotropy. Related theoretical studies only considered bulk conditions and thus neglected the influence from possible strain linked to the choice of substrate. Here we employ first principles calculations considering different exchange–correlation functionals (PBE, PW91, PBEsol, AM05, LDA) and effect from use of + U methods (or not) combined with a magnetic ground-state search using Heisenberg Monte Carlo simulations, to study influence from biaxial in-plane strain and external pressure on the magnetic and crystal structure of Mn_2_GaC. We find that PBE and PBE + U, with *U*_eff_ ≤ 0.25 eV, gives both structural and magnetic properties in quantitative agreement with available experimental data. Our results also indicate that strain related to choice of substrate or applied pressure is a route for accessing different spin configurations, including a ferromagnetic state. Moreover, the easy axis is parallel to the atomic planes and the magnetocrystalline anisotropy energy can be increased through strain engineering by expanding the in-plane lattice parameter *a*. Altogether, we show that a quantitative description of the structural and magnetic properties of Mn_2_GaC is possible using PBE, which opens the way for further computational studies of these and related materials.

## Introduction

In the 1960s, Nowotny and coworkers^1,2^ discovered a family of inherently atomically laminated materials, which decades later were coined MAX phases. It is a ternary phase with the general formula *M*_*n*+1_*AX*_*n*_ (*n* = 1–3) where *M* is a transition metal (e.g. *M* = Ti, Cr, Mo, Zr), *A* is typically a group 13 to 16 element (e.g. *A* = Al, Si, Ga, Ge), and *X* is carbon or nitrogen. The material family, however, did not receive much attention until the mid-1990s and early 2000s when Ti_3_SiC_2_^3^ and Ti_4_AlN_3_^4,5^, respectively, was demonstrated to possess a unique combination of metallic and ceramic characteristics. Since then, MAX phases have been synthesized both as bulk material and in thin film form, and have been shown to exhibit extraordinary physical, chemical, electrical and mechanical properties^6^. Due to this, the MAX phases are being considered for protective coatings, electrical contacts, sensors, and high-temperature structural applications. They are also used as precursors for MXenes, their two-dimensional (2D) counterparts exhibiting extraordinary properties and are considered for a host of different applications^7–9^.

The first experimental evidence of magnetic MAX phases were quaternary Mn-doped Cr_2_GeC^10–12^, Cr_2_GaC^13–17^ and Cr_2_AlC^14,18,19^ followed by (Mo,Mn)_2_GaC^20,21^ and (V,Mn)_3_GaC_2_^22^. Examples of ternary MAX phases with demonstrated magnetic properties are Cr_2_AlC^23,24^, Cr_2_GeC^11,24^, Cr_2_GaC^13,25^, Cr_2_GaN^25^, and Mn_2_GaC^26–29^. The Cr-based phases have all been made in bulk form and as thin films, whereas Mn_2_GaC have been synthesized in thin film form only. Potential applications of magnetic MAX phases range from spintronics to refrigeration, even though the research efforts have so far been focused solely on the discovery of new magnetic phases and compositions, and fundamentals of magnetic properties as summarized in Ref.^30^.

It has previously been shown that Mn_2_GaC simultaneously undergoes a magnetic and structural transition between 230 and 250 K^27^. With decreased temperature, the out-of-plane *c* axis contracts by 0.2% with an asymmetric change of the crystal structure as demonstrated from both X-ray diffraction^27^ and neutron diffraction^28^. It has also been shown that at 214 K Mn_2_GaC undergoes a first order magnetic phase transition from antiferromagnetic (AFM) at higher temperature to a non-collinear AFM spin structure with a large uniaxial *c*-axis magnetostriction of 450 ppm^29^. Moreover, Mn_2_GaC exhibits neutron-diffraction peaks consistent with long-range AFM order with a periodicity of two structural unit cells^28^. Also, a local magnetic moment of ~ 1.7 µ_B_ per Mn atom at 3 K and 5 T have been measured^27^. Based on supercell calculations, the magnetic critical order–disorder temperature T_c_ has been predicted to be 660 ± 133K^31^. Subsequent measurement found a Néel temperature of 507 K, at which Mn_2_GaC changes from a (suggested) collinear AFM state to the paramagnetic (PM) state^29^.

The need for using DFT + U methods for studying magnetic MAX phases have been debated, see Ref. ^30^ for further details. The first studies motivate the use of + U to get a better correlation between measured and calculated bulk modulus for Cr_2_*A*C (*A* = Al, Ga, Ge)^32–37^. We have later shown that a better match can be achieved without any + U but through extended unit cells to describe non-trivial magnetic configurations^38,39^. Most investigations using + U for studying magnetic MAX phases use *U* from 0 to 2 eV, while, e.g., the hypothetical 2D Mn_2_C MXene was studied using *U* = 4 eV and *J* = 1 eV, motivated by studies of Mn-based 3D compounds such as La(Mn,Zn)AsO, (Ga,Mn)N^40^.

For the comparatively unexplored (theoretically and experimentally) Mn_2_GaC phase, it is therefore motivated to investigate if the use of the ordinary generalized gradient approximation (GGA) is enough to describe the electron correlation, or if other functionals or DFT + U methods are necessary to describe the material. To address these questions, we here chose to investigate the structure and magnetic characteristics of Mn_2_GaC under thin film constraints from choice of substrate, by applying tensile and compressive strain, both in- and out-of-plane, while considering different exchange–correlation functionals as well as use of DFT + U methods. In addition, we explore the easy axis and elaborate on its correlation to applied strain.

## Result and discussion

In previous work of Mn_2_GaC, we presented theoretical results valid for bulk synthesis conditions and thermodynamic equilibrium, by energies and volumes for various spin configurations obtained through complete relaxation of the unit cell volume, lattice parameters and atomic positions using the PBE functional^26–28,31^. Thin films, on the other hand, are known to realize also metastable states, sometimes far from thermodynamic equilibrium, with the lattice parameter of the film material influenced by the choice of substrate. Since Mn_2_GaC has been synthesized as epitaxial thin films only, we here use the approach of performing structural relaxation under constraints that mimic thin film conditions. Depending on the substrate used, different lattice parameters may be achieved due to strain, which in turn may influence the magnetic properties.

**Figure 1:**
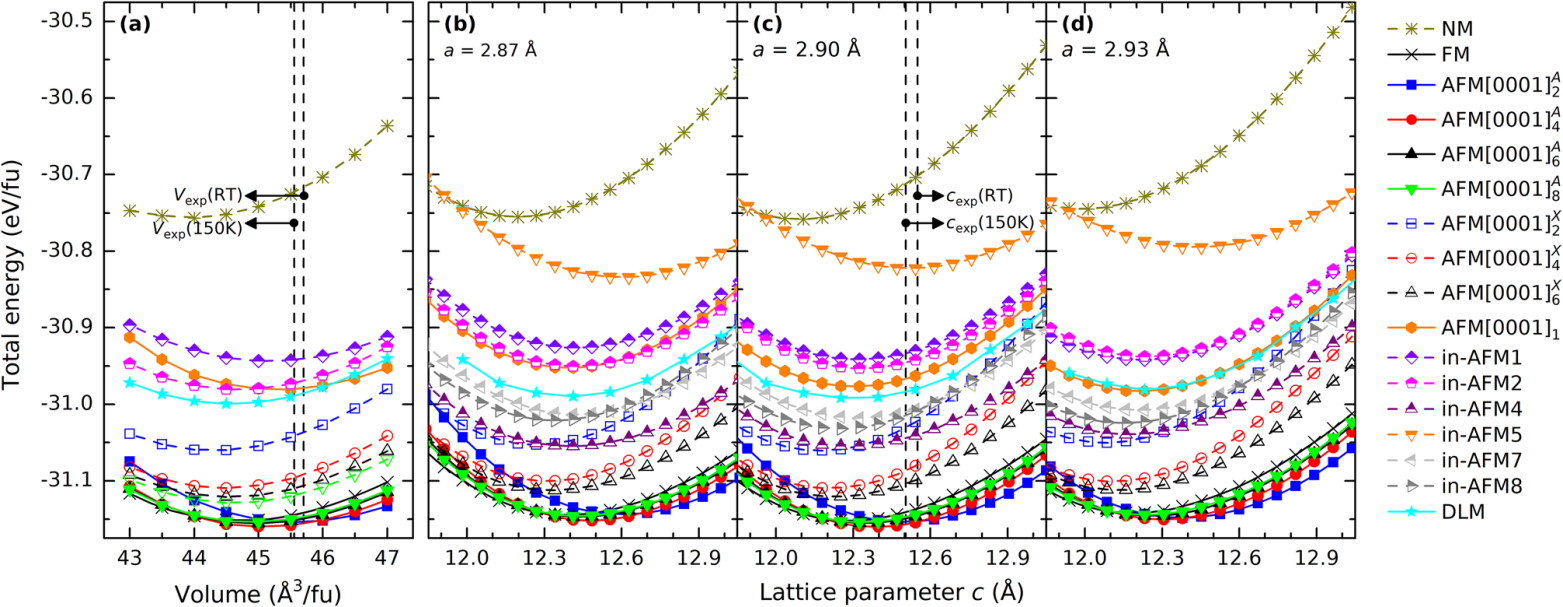
Total energy as a function of **(a)** volume and out-of-plane lattice parameter *c* for in-plane lattice parameter *a* being **(b)** 2.87 Å, **(c)** 2.90 Å, and **(d)** 2.93 Å assuming different spin configurations. All data presented are based on first-principles calculations employing the PBE exchange–correlation functional. The experimentally measured volume and lattice parameter *c* at room temperature (RT) and 150 K is represented by the vertical dashed line^26,27^.

As a first step, we include collinear spin configurations defined in Fig. 8 in Computational Details and in Table S1, using the PBE functional. We relax the structure (i) as function of volume and (ii) under the constraint of keeping the in-plane lattice parameter *a* fixed (at compressed (*a* = 2.87), equilibrium (*a*_0_ = 2.90 Å), and tensile (*a* = 2.93 Å) strains) while changing the out-of-plane lattice parameter *c*. Figure 1 show the rather large spread in energy for the various spin configurations. For both volume relaxation and with Mn_2_GaC constrained in-plane we find qualitatively similar results, with FM and ${\text{AFM}}{\left[0001\right]}_{\alpha }^{A}$ being lowest in energy. Optimized lattice parameters for considered collinear spin configurations are found in Table 1.

**Table 1:** Calculated equilibrium volume *V*, lattice parameters *a* and *c*, and absolute magnetic moment per Mn atom for considered magnetic spin configurations of Mn_2_GaC using the PBE exchange–correlation functional.

Magnetic state	Structural parameters			Local magnetic moments
	*V* (Å^3^/fu)	*a* (Å)	*c* (Å)	|m| (${\mu }_{B}$/Mn atom)
${\text{NM}}$	43.90	2.892	12.13	–
${\text{FM}}$	44.72	2.899	12.29	1.95
${\text{AFM}}{\left[0001\right]}_{2}^{A}$	45.57	2.903	12.48	2.26
${\text{AFM}}{\left[0001\right]}_{4}^{A}$	45.07	2.898	12.39	1.99, 2.17
${\text{AFM}}{\left[0001\right]}_{6}^{A}$	44.96	2.900	12.34	1.92, 2.01, 2.17
${\text{AFM}}{\left[0001\right]}_{8}^{A}$	44.89	2.902	12.31	1.94, 1.97, 2.00, 2.16
${\text{AFM}}{\left[0001\right]}_{2}^{X}$	44.29	2.898	12.18	1.59
${\text{AFM}}{\left[0001\right]}_{4}^{X}$	44.46	2.900	12.21	1.52, 1.95
${\text{AFM}}{\left[0001\right]}_{6}^{X}$	44.55	2.899	12.24	1.54, 1.91, 1.97
${\text{AFM}}{\left[0001\right]}_{8}^{X}$	44.60	2.899	12.26	1.54, 1.94, 1.96, 1.97
${\text{AFM}}{\left[0001\right]}_{1}$	45.31	2.925	12.23	1.83
In-${\text{AFM1}}$	45.15	2.916	12.27	1.95
In-${\text{AFM2}}$	44.67	2.892	12.35	1.99
In-AFM4	44.68	2.890	12.35	1.83, 1.95
In-AFM5	44.77	2.869	12.58	1.88
In-AFM7	44.79	2.895	12.34	1.79, 1.88, 2.05
In-AFM8	44.63	2.900	12.24	1.63, 1.86
DLM	44.67	2.887	12.38	0.57 to 2.27
Exp.^a^	45.70	2.90	12.55	Not reported
Exp.^b^	45.56	2.90	12.51	1.7 (at 3 K and 5 T)

Experimental results are included for comparison.

^a^Structural parameters measured at room temperature^26^.

^b^Structural parameters measured at 150 K^27^.

Based on Fig. 1 the focus will from this point forward be on magnetic spin configurations in the low-energy region and investigate how biaxial in-plane strain, i.e., effect from using different substrates, and pressure applied perpendicular to the film surface, i.e., along the surface normal (the *c*-axis), may alter the crystal structure and magnetic properties of Mn_2_GaC. In addition, we also include a magnetic ground-state search at different biaxial strains and look at the magnetic anisotropy energies. Moreover, we herein also evaluate the effect from choice of different exchange correlation functionals.

### Biaxial in-plane strain

We first investigate the impact from biaxial in-plane strain on the magnetic and the crystal structure of Mn_2_GaC using PBE, PW91, PBEsol, AM05, and LDA functionals. We consider seven representative spin configurations (FM, $\mathrm{A}\mathrm{F}\mathrm{M}{\left[0001\right]}_{2}^{A}$, $\mathrm{A}\mathrm{F}\mathrm{M}{\left[0001\right]}_{4}^{A}$, $\mathrm{A}\mathrm{F}\mathrm{M}{\left[0001\right]}_{1}$, $\mathrm{A}\mathrm{F}\mathrm{M}{\left[0001\right]}_{2}^{X}$, $\mathrm{A}\mathrm{F}\mathrm{M}{\left[0001\right]}_{4}^{X}$, and in-AFM2) along with the non-magnetic (NM) solution, at various strains. Independent of strain or functional used we find that the overall low-energy spin configurations are FM, $\mathrm{A}\mathrm{F}\mathrm{M}{\left[0001\right]}_{2}^{A}$, and $\mathrm{A}\mathrm{F}\mathrm{M}{\left[0001\right]}_{4}^{A}$, as shown in Fig. S1. These three spin configurations are therefore chosen for further evaluation.

**Figure 2:**
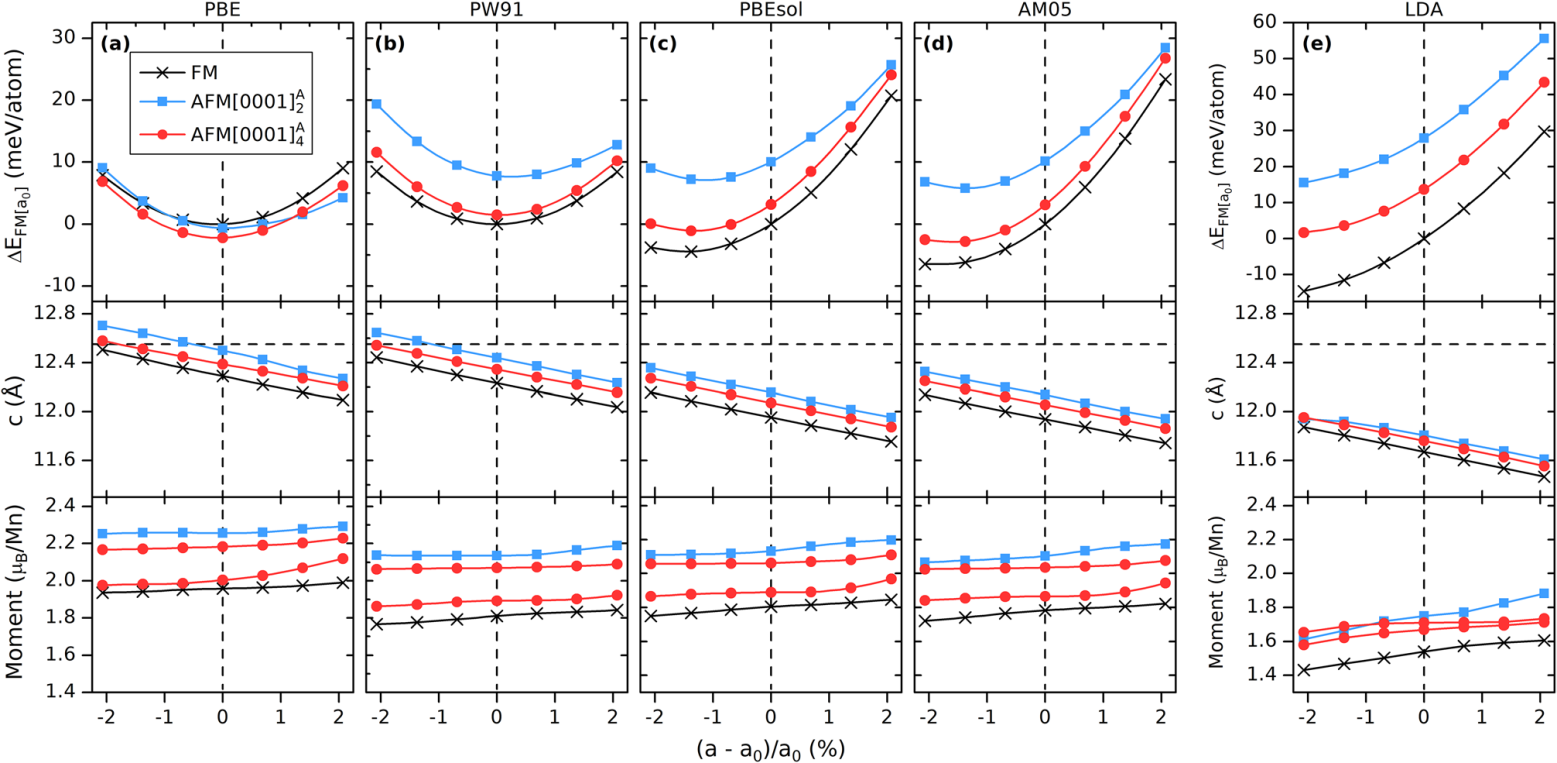
Energy relative to the FM state with *a*_0_ = 2.90 Å (upper panels), lattice parameter c (mid panels), and local magnetic moment (bottom panels) as function of biaxial in-plane strain for FM, $\mathrm{A}\mathrm{F}\mathrm{M}{\left[0001\right]}_{2}^{A}$, and $\mathrm{A}\mathrm{F}\mathrm{M}{\left[0001\right]}_{4}^{A}$ spin configurations using **(a)** PBE, **(b)** PW91, **(c)** PBEsol, **(d)** AM05, and **(e)** LDA exchange–correlation functionals. The experimentally measured lattice parameter *a* of 2.90 Å is represented by the vertical dashed line and *c* of 12.55 Å is represented by the horizontal dashed line in the mid panels^26, 27^.

In Fig. 2 the energy difference ΔE relative to a FM state, with *a*_0_ = 2.90 Å, and local magnetic moments as function of biaxial in-plane strain is shown for FM, $\mathrm{A}\mathrm{F}\mathrm{M}{\left[0001\right]}_{2}^{A}$, and $\mathrm{A}\mathrm{F}\mathrm{M}{\left[0001\right]}_{4}^{A}$. Looking at ΔE in the top panels in Fig. 2, four functionals, PW91, LDA, PBEsol, and AM05, show the same spin configuration order with FM lowest in energy, $\mathrm{A}\mathrm{F}\mathrm{M}{\left[0001\right]}_{2}^{A}$ highest in energy, and $\mathrm{A}\mathrm{F}\mathrm{M}{\left[0001\right]}_{4}^{A}$ in-between. For PBE, $\mathrm{A}\mathrm{F}\mathrm{M}{\left[0001\right]}_{4}^{A}$ is lowest in energy at compressive strains and up to + 1% tensile strain. Above + 1.3%, $\mathrm{A}\mathrm{F}\mathrm{M}{\left[0001\right]}_{2}^{A}$ is found to be of lowest energy. Only two functionals, PBE and PW91, results in an energy minimum at or close the reported experimental value of *a* = 2.90 Å. LDA, PBEsol, and AM05 all overbind with a minimum ΔE at compressive strains below − 1.5%.

From a structural point of view, we find a decrease in lattice parameter *c* with increasing *a*, see the mid panels in Fig. 2. For all functionals we find *c* to be smallest for FM and largest for $\mathrm{A}\mathrm{F}\mathrm{M}{\left[0001\right]}_{2}^{A}$. The reported value of *c* (12.55 Å) for synthesized Mn_2_GaC, with *a* = 2.90 Å, is indicated by the dashed horizontal line. Both PBE and PW91 give *c* values close to reported, while overbinding (resulting in too small *c*) is large for PBEsol, AM05, and especially LDA.

Calculated local moments, bottom panels of Fig. 2, are in general highest for $\mathrm{A}\mathrm{F}\mathrm{M}{\left[0001\right]}_{2}^{A}$ and lowest for FM. For $\mathrm{A}\mathrm{F}\mathrm{M}{\left[0001\right]}_{4}^{A}$ we observe two different values which can be related to its spin structure that on a local scale resembles both FM and $\mathrm{A}\mathrm{F}\mathrm{M}{\left[0001\right]}_{2}^{A}$. This can be seen by comparing the magnetic structures in Fig. 7d with Fig. 6a, c in Computational Details, where the lower value corresponds to a FM surrounding, with Mn atoms of parallel spins across the Ga layer, while those of higher value resemble $\mathrm{A}\mathrm{F}\mathrm{M}{\left[0001\right]}_{2}^{A}$, with Mn atoms of antiparallel spins across the Ga layer.

The internal crystal structure of Mn_2_GaC can be described by two different Mn–Mn interlayer distances illustrated in Fig. 8q in Computational Details; (i) *d*_*X*_ which represents the distance between two Mn layers interleaved with carbon, or *X* in general, and (ii) *d*_*A*_ which represents the distance between two Mn layers interleaved with Ga, or *A* in general. Fig. S2 show interlayer distances *d*_*X*_ and *d*_*A*_ as function of biaxial in-plane strain. For $\mathrm{A}\mathrm{F}\mathrm{M}{\left[0001\right]}_{4}^{A}$ there are two different *d*_*A*_ values which can be related to the difference in the spin configuration close to the Mn layer. Similar to the two different magnetic moments in Fig. 2. The lower *d*_*A*_ value is similar to those found for FM, as are their local moments, while the larger *d*_*A*_ value is close to those obtained for $\mathrm{A}\mathrm{F}\mathrm{M}{\left[0001\right]}_{2}^{A}$. For *d*_*X*_, only one set of values is found for each of the three spin configurations, and these configurations are, on a local scale, equivalent.

**Figure 3:**
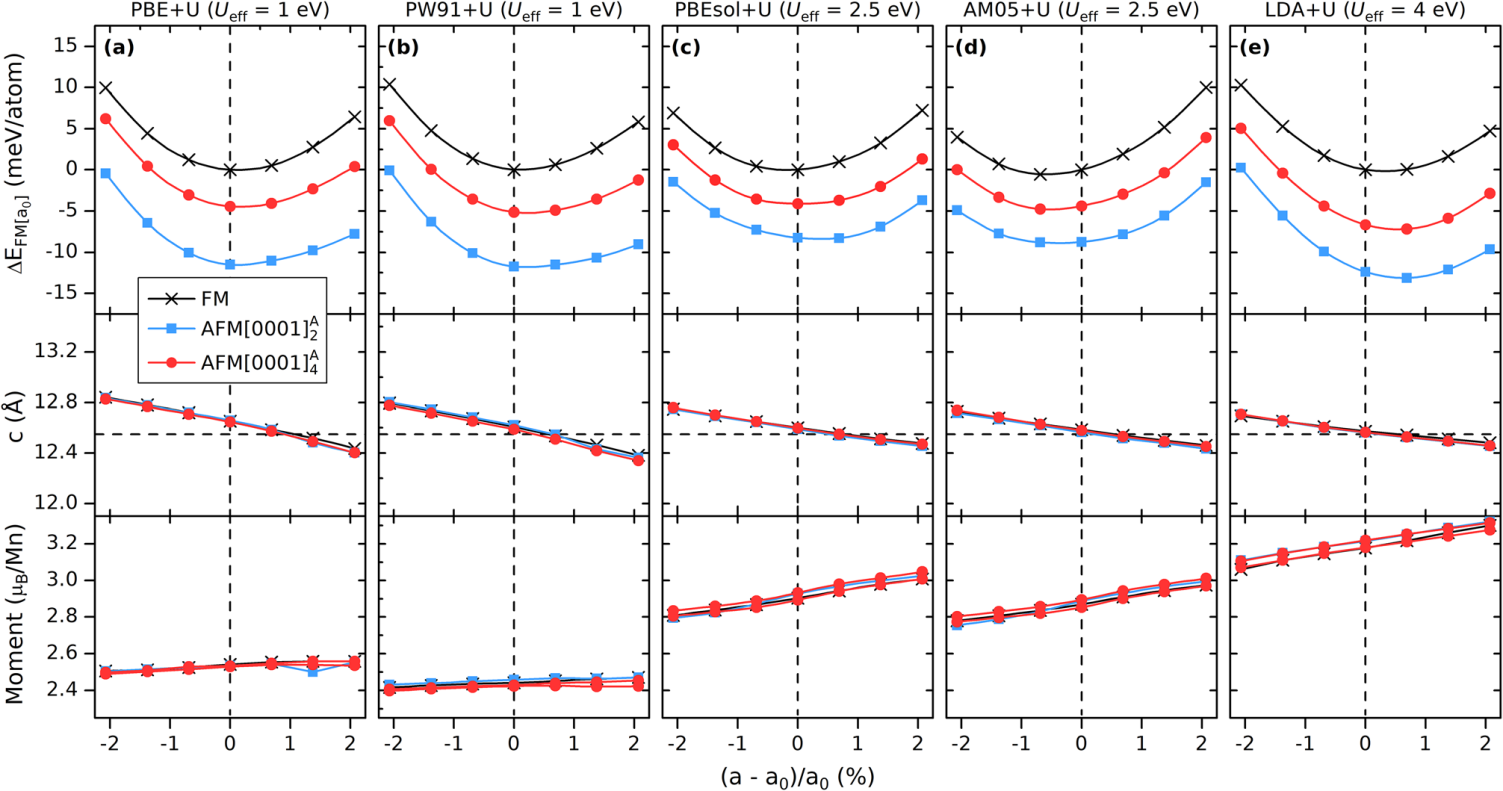
Energy relative ferromagnetic solution with *a*_0_ = 2.90 Å, Δ*E* (top panels), and local magnetic moment (bottom panels) as function of strain for FM, $\mathrm{A}\mathrm{F}\mathrm{M}{\left[0001\right]}_{2}^{A}$, and $\mathrm{A}\mathrm{F}\mathrm{M}{\left[0001\right]}_{4}^{A}$ spin configurations using **(a)** PBE + U, **(b)** PW91 + U, **(c)** PBEsol + U, **(d)** AM05 + U, and **(e)** LDA + U exchange–correlation functionals. Considered *U*_eff_ values are given at the top of each panel. The experimentally measured lattice parameter *a* of 2.90 Å is represented by the vertical dashed line and *c* of 12.55 Å is represented by the horizontal dashed line in the mid panels^26,27^.

Based on the large tendencies for overbinding when using PBEsol, AM05, and LDA functionals, we chose to investigate impact from biaxial strain upon addition of + U, using the approach by Dudarev^41^. It should be noted that in a theoretical study of the hypothetical 2D Mn_2_C MXene^40^, which is the 2D counterpart of Mn_2_GaC after removal of Ga, *U* = 4 eV and *J* = 1 eV was used. Such value of *U* can not directly be extrapolated to also be valid for the 3D Mn_2_GaC. We have therefore used several values for *U*_eff_, though for the results presented herein, *U*_eff_ is chosen based on an identified minimum ΔE close to *a*_0_ = 2.90 Å and values for *c* close to the reported value of 12.55 Å. A moderate value of *U*_eff_ = 1 eV is chosen for PBE and PW91 as their numbers are already close to or slightly larger than reported experimental values^26,27^. Both PBEsol and AM05 require *U*_eff_ = 2.5 eV, while for LDA a value of *U*_eff_ = 4 eV is needed. To ensure that no other spin configurations than FM, $\mathrm{A}\mathrm{F}\mathrm{M}{\left[0001\right]}_{2}^{A}$, and $\mathrm{A}\mathrm{F}\mathrm{M}{\left[0001\right]}_{4}^{A}$ may be a relevant low-energy solution, we also consider $\mathrm{A}\mathrm{F}\mathrm{M}{\left[0001\right]}_{1}$, $\mathrm{A}\mathrm{F}\mathrm{M}{\left[0001\right]}_{2}^{X}$, $\mathrm{A}\mathrm{F}\mathrm{M}{\left[0001\right]}_{4}^{X}$, in-AFM2, and NM. As shown in Fig. S3, the low-energy spin configurations are those with parallel spins within each Mn-C-Mn trilayer, i.e., FM, $\mathrm{A}\mathrm{F}\mathrm{M}{\left[0001\right]}_{2}^{A}$, and $\mathrm{A}\mathrm{F}\mathrm{M}{\left[0001\right]}_{4}^{A}$. Interesting to note is that for all six functionals we find the order of the spin configurations to be the same, with $\mathrm{A}\mathrm{F}\mathrm{M}{\left[0001\right]}_{2}^{A}$ lowest in energy followed by $\mathrm{A}\mathrm{F}\mathrm{M}{\left[0001\right]}_{4}^{A}$ and FM as seen in Fig. 3. Moreover, for $\mathrm{A}\mathrm{F}\mathrm{M}{\left[0001\right]}_{4}^{A}$ in Fig. 3 and Fig. S4 there is no longer two distinct values of neither the local magnetic moment or *d*_A_, as compared to Fig. 2 and Fig. S4, when + U is not considered. Instead, these values are similar to values found for both FM and $\mathrm{A}\mathrm{F}\mathrm{M}{\left[0001\right]}_{2}^{A}$.

The results presented for Mn_2_GaC at biaxial strain shows that PBEsol, AM05, and LDA all overbind and with FM as the lowest energy spin configuration. PBE and PW91, on the other hand, results in an energy minimum at or close the reported experimental value of *a* = 2.90 Å and with magnetic moments slightly overestimated than experimentally reported^27^. The use of + U, with *U*_eff_ values chosen in such a way to mimic experimentally reported lattice parameters, results in local magnetic moments much larger than those reported experimentally^27^. We also note, independent of functional or if using + U or not, that the corresponding low-energy spin configurations always corresponds to those with FM ordering within Mn-C-Mn trilayer, i.e., FM and ${\text{AFM}}{\left[0001\right]}_{\alpha }^{A}$ (α = 2 and 4). Such FM ordering within Mn-C-Mn trilayer has previously been experimentally demonstrated for quaternary MAX phases (Cr_0.5_Mn_0.5_)_2_GaC^15,16^ and (Mo_0.5_Mn_0.5_)_2_GaC^20,21^ using ferromagnetic resonance (FMR) measurements. We thus conclude that using PBE, without + U or a with a small *U*_eff_, gives crystallographic structures, including the asymmetric structural changes during the magnetic transition around 250 K and below^27^, and magnetic structures comparable to those found from vibrating sampling magnetometry, X-ray and neutron diffraction ^26–28^. Note that the use of no U or a small value of *U*_eff_ for 3D Mn_2_GaC, as compared to *U* = 4 eV and *J* = 1 eV used for 2D Mn_2_C^40^, could be related to the interference by the Ga layer, imposing periodic screening of the Mn-C-Mn trilayers.

### Magnetic ground-state search at biaxial in-plane strain

To this point, only collinear spin configurations have been considered. From analysis of magnetic measurements of Mn_2_GaC there are indications that the magnetic structure is, or at least bears strong resemblance to, AFM ${\left[0001\right]}_{4}^{A}$. One approach to identify or get inspiration for other possible and more complex noncollinear spin configurations is to perform a representative magnetic ground-state search. In Ref.^27^ such a search was performed and several magnetic spin configuration close to degenerate in energy was found as a function of volume.

Motivated by the thin films of Mn_2_GaC synthesized to date, a representative magnetic ground-state search needs to keep *a* static to mimic thin film conditions for different substrates. We used the same approach as in Ref.^27^ and performed a magnetic ground-state search using a coarse-grained super-moment representation, depicted in Fig. 9 in Computational Details, aiming to find additional magnetic spin configurations. To preserve control of the crystal structure, and the corresponding energies, we made the following approximations;

(i) *a* and interlayer distance *d*_*X*_ were kept fixed while varying the interlayer distance *d*_*A*_.
(ii) we only considered low-energy candidates with parallel spins within the Mn-C-Mn trilayer, i.e., FM, $\mathrm{A}\mathrm{F}\mathrm{M}{\left[0001\right]}_{2}^{A}$, $\mathrm{A}\mathrm{F}\mathrm{M}{\left[0001\right]}_{4}^{A}$, $\mathrm{A}\mathrm{F}\mathrm{M}{\left[0001\right]}_{6}^{A}$ and $\mathrm{A}\mathrm{F}\mathrm{M}{\left[0001\right]}_{8}^{A}$.
(iii) we used the PBE exchange–correlation functional with no + U.

Based on these constraints we calculated the total energies, shown in Fig. S5, and used these along with the Connolly-Williams structure inversion method^42,43^ to derive the exchange interactions *J*_ij_’s for the first four super-moment interlayer coordination shells, see Fig. S6. In order to find possible long-range magnetic spin configurations, we considered supermoment chains with up to 40 beads. This range also ensures that we minimize effects related to size and boundary conditions. The energy dependence of the number of beads included in the chain at various values of *d*_*A*_ is shown in Fig. S8. From this point forward, we only present the low-energy solutions for each set of *a*, *d*_*X*_, and *d*_*A*_, independent on the number of beads used.

**Figure 4:**
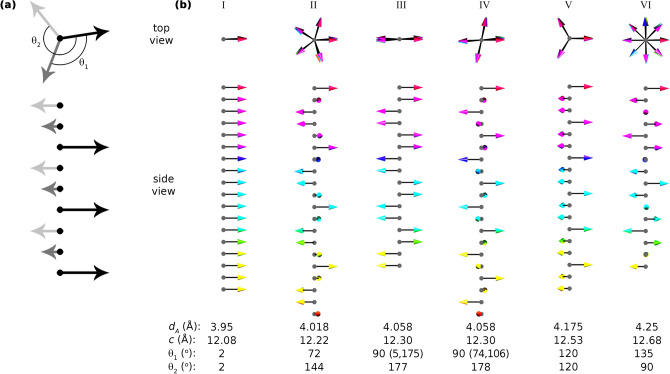
**(a)** Schematic illustration individual angles defining the spin-orientation between nearest and next nearest neighbour supermoment spin vectors, θ_1_ and θ_2_. **(b)** Selected low-energy spin configurations extracted from the Heisenberg Monte Carlo simulations at various *d*_*A*_ where *a* = 2.90 Å and *d*_*X*_ = 2.09 Å.

Figure 4 summarizes the result from our Heisenberg Monte Carlo simulations. Figure 4a shows a schematic illustration of the two individual angles defining the spin-orientation between nearest and next nearest neighbour supermoment spin vectors, θ_1_ and θ_2_. Details of θ_1_ and θ_2_ extracted from low-energy solutions when varying *a*, *d*_*X*_, and *d*_*A*_ is shown in Fig. S8. A selection of representative low-energy solutions is illustrated in Fig. 4b along with related information such as interlayer distance *d*_*A*_, corresponding lattice parameter *c*, and average and/or individual angle between nearest and next nearest neighbour supermoment spin vectors, ${\stackrel{-}{\uptheta }}_{1}$ and ${\stackrel{-}{\uptheta }}_{2}$. Note that for *d*_*A*_ = 4.058 Å (*c* = 12.30 Å), two different solutions were found with equal energy, III and IV, each with two distinct values of ${\uptheta }_{1}$, as given within parenthesis, though with an average value of $90^\circ$. Similar results are found when Mn_2_GaC is under compressive (-1%, *a* = 2.87 Å) and tensile (+ 1%, *a* = 2.93 Å) strain, shown in Fig. S8, although shifted to larger and smaller *d*_*A*_, respectively. When smaller(larger) values of *d*_*X*_ are considered, the results are shifted to larger(smaller) value of *d*_*A*_, shown in Fig. S8.

**Figure 5:**
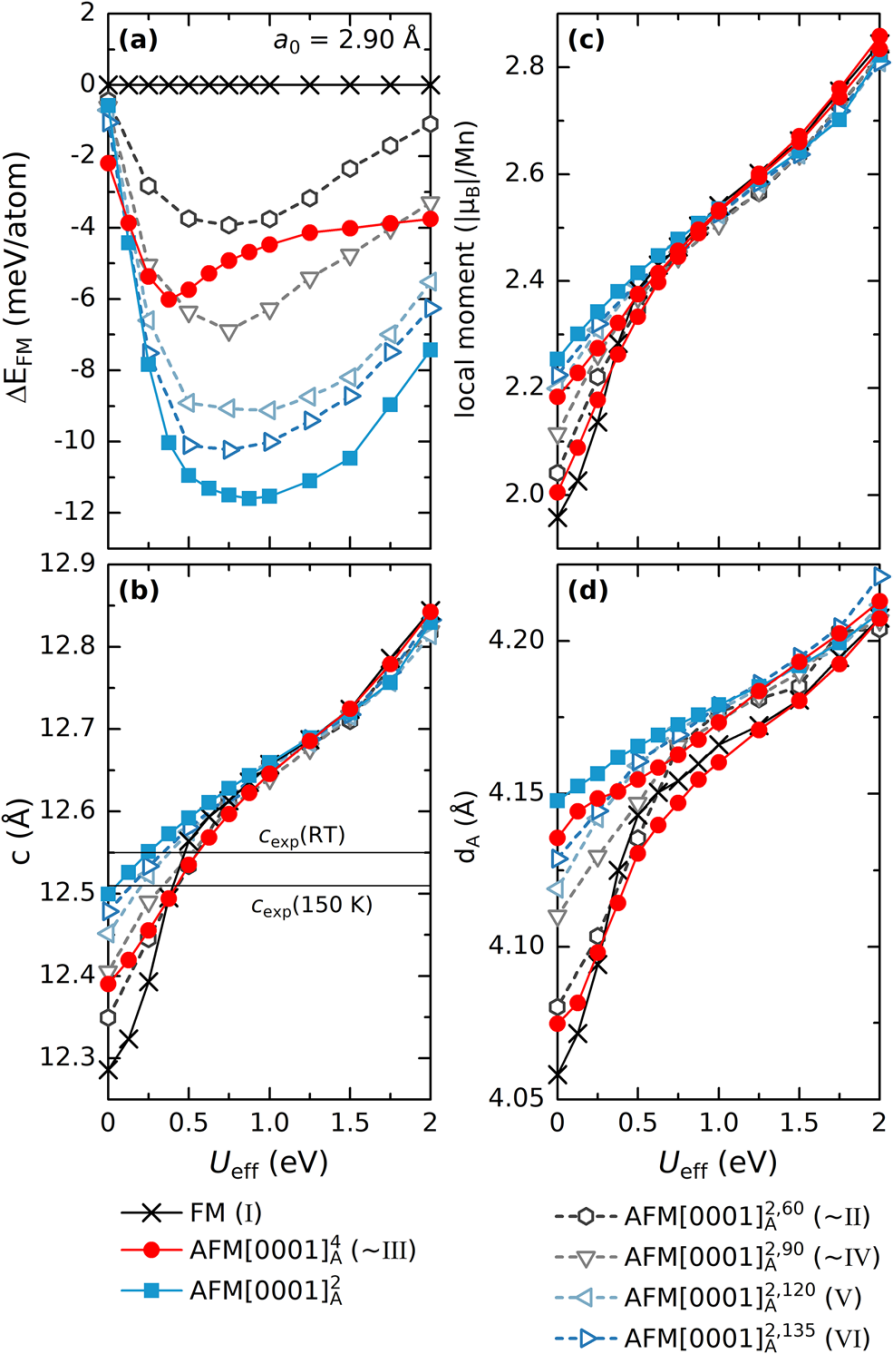
**(a)** Energy relative to the FM state, **(b)** lattice parameter *c*, **(c)** local magnetic moment, and **(d)** interlayer distances *d*_*A*_ for Mn_2_GaC, with *a*_0_ = 2.90, Å as function *U*_eff_ for collinear FM, $\mathrm{A}\mathrm{F}\mathrm{M}{\left[0001\right]}_{2}^{A}$, and $\mathrm{A}\mathrm{F}\mathrm{M}{\left[0001\right]}_{4}^{A}$ and noncollinerar $\mathrm{A}\mathrm{F}\mathrm{M}{\left[0001\right]}_{2}^{A,60}$, $\mathrm{A}\mathrm{F}\mathrm{M}{\left[0001\right]}_{2}^{A,90}$, $\mathrm{A}\mathrm{F}\mathrm{M}{\left[0001\right]}_{2}^{A,120}$, and $\mathrm{A}\mathrm{F}\mathrm{M}{\left[0001\right]}_{2}^{A,135}$ spin configurations using the PBE + U exchange–correlation functional.

Inspired by the obtained noncollinear spin spiral configurations depicted in Fig. 4b, we considered a small set of representative configurations for evaluation using first-principles calculations. For *a*_0_ = 2.90 Å, we use PBE + U with *U*_eff_ from 0 to 2 eV and compare energies, structural parameters and magnetic moments, see Fig. 5. The calculated energies relative to the FM state is shown in in Fig. 5a. With ordinary PBE (*U*_eff_ = 0 eV), the considered noncollinear spin configurations are all in between FM and the lowest energy $\mathrm{A}\mathrm{F}\mathrm{M}{\left[0001\right]}_{4}^{A}$. For PBE + U, with increasing *U*_eff_ up to 0.5 eV, all AFM configurations, collinear and noncollinear, decreases in energy relative to FM.

Measured lattice parameter *c* at room temperature (RT) and at 150 K, indicated by horizontal lines in Fig. 5b, clearly shows that using ordinary PBE gives too small *c* while *U*_eff_ ≥ 0.5 eV gives too large values of *c*. Moreover, not only does *c*, the interlayer distance *d*_*A*_ and the local magnetic moment increase with increasing *U*_eff_, but we also find that they merge towards similar values independent of considered spin configuration. This is most clear for *U*_eff_ ≥ 1 eV. This is indicative of a parameter forcing the magnetic moment to be localized, raising a question of the proper value of *U*_eff_ to describe Mn_2_GaC in particular and magnetic MAX phases in general. These results indicate the PBE or PBE + U (with *U*_eff_ ≤ 0.25 eV) is the most appropriate functionals to use until proven otherwise for describing Mn_2_GaC when compared to experimental results^26–29^.

### Mn_2_GaC under pressure

Here we investigate how the magnetic properties and crystal structure of Mn_2_GaC can be altered when pressure is applied perpendicular to the film surface, i.e., along the surface normal (the *c*-axis). We only consider low energy spin configurations FM, $\mathrm{A}\mathrm{F}\mathrm{M}{\left[0001\right]}_{2}^{A}$, and $\mathrm{A}\mathrm{F}\mathrm{M}{\left[0001\right]}_{4}^{A}$, and choose to not include noncollinear spin configurations since the energies for these are all found in between those of FM, $\mathrm{A}\mathrm{F}\mathrm{M}{\left[0001\right]}_{2}^{A}$ and $\mathrm{A}\mathrm{F}\mathrm{M}{\left[0001\right]}_{4}^{A}.$ Starting with the thermodynamic stability of Mn_2_GaC, the possibility of phase transition between considered spin configurations is investigated. The enthalpies are calculated according to

1 $$H\left[p\right]=E\left[p\right]+pV\left[p\right],$$

where *E*[*p*] and *V*[*p*] is the equilibrium energy and volume, respectively, at given pressure *p*.

**Figure 6:**
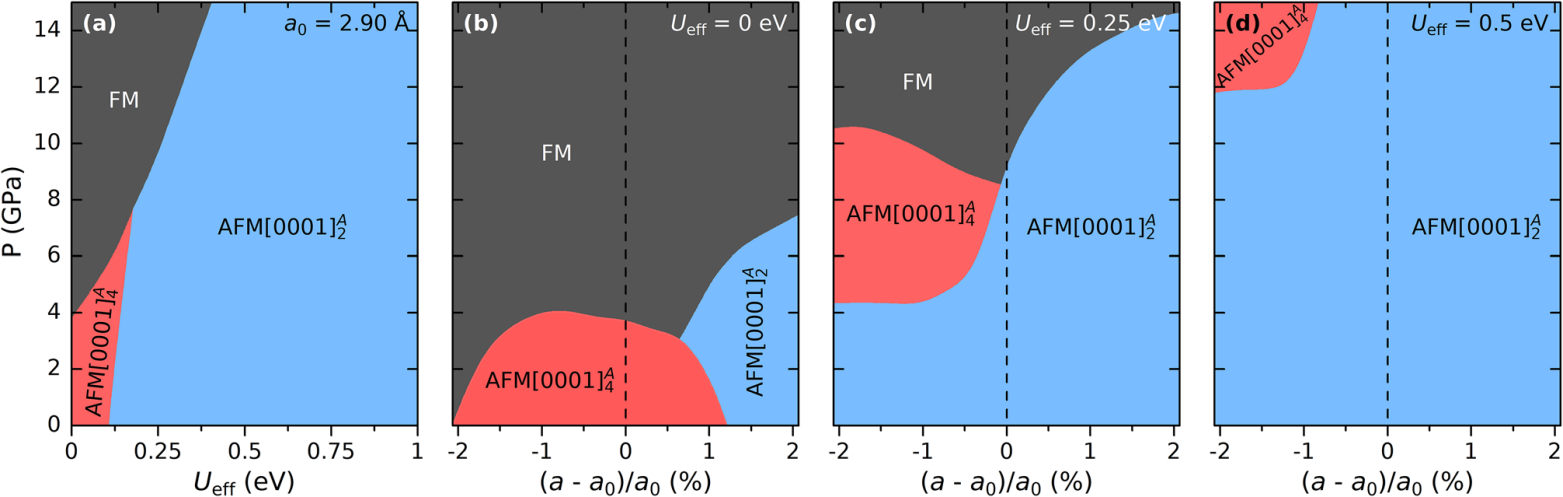
Spin configuration of lowest enthalpy for **(a)** a pressure vs *U*_eff_ grid, and for a pressure vs biaxial-in-plane strain grid for *U*_eff_ equal to **(b)** 0 eV, **(c)** 0.25 eV, and **(d)** 0.5 eV, using the PBE + U exchange–correlation functional.

Figure 6a depicts the pressure vs *U*_eff_ where the color represents the spin configuration of lowest enthalpy for *a*_0_ = 2.90 Å. For ordinary PBE (*U*_eff_ = 0) we find that at 3.8 GPa there is a transition from $\mathrm{A}\mathrm{F}\mathrm{M}{\left[0001\right]}_{4}^{A}$ to FM. When *U*_eff_ increases $\mathrm{A}\mathrm{F}\mathrm{M}{\left[0001\right]}_{2}^{A}$ becomes the favoured configuration at small applied pressures. FM is still accessible for *U*_eff_ > 0 but an increased pressure is required.

To explore potential pathways for attaining a FM state, we also probe for lowest enthalpy spin configuration on a pressure vs biaxial-in-plane strain grid for a range of lattice parameter *a,* from compressive (*a* = 2.86 Å, − 2.07%) to tensile (*a* = 2.94 Å, + 2.07%) strain. The various values of *a* thus represent substrates of different size. Figure 6b depicts pressure vs bi-axial in-plane strain for ordinary PBE (*U*_eff_ = 0 eV) where the color represents the spin configuration of lowest enthalpy. At zero pressure and from compressive up to 1.25% tensile biaxial strain, we find $\mathrm{A}\mathrm{F}\mathrm{M}{\left[0001\right]}_{4}^{A}$, while further increase in tensile strain results in a transition to $\mathrm{A}\mathrm{F}\mathrm{M}{\left[0001\right]}_{2}^{A}$. When pressure is applied, the enthalpy difference between $\mathrm{A}\mathrm{F}\mathrm{M}{\left[0001\right]}_{\alpha }^{A}$ and FM decreases. For *a*_0_ = 2.90 Å (0%) we find that at 3.8 GPa there is a transition from $\mathrm{A}\mathrm{F}\mathrm{M}{\left[0001\right]}_{4}^{A}$ to FM. This transition is associated with a corresponding decrease in *c*, magnetic moment, and interlayer distances *d*_*A*_ and *d*_*X*_, see details in Fig. S9, and is found at both compressive (down to -2%) and tensile (up to + 1.25%) biaxial strain for an applied pressure up to 4 GPa. Between tensile strain from + 0.75 to 1.25% there is a transition from $\mathrm{A}\mathrm{F}\mathrm{M}{\left[0001\right]}_{4}^{A}$ to $\mathrm{A}\mathrm{F}\mathrm{M}{\left[0001\right]}_{2}^{A}$ with increasing pressure before accessing FM. Furthermore, for a tensile biaxial strain above + 1.25% and at zero or low pressure we find $\mathrm{A}\mathrm{F}\mathrm{M}{\left[0001\right]}_{2}^{A}$. When pressure is applied there is a transition from $\mathrm{A}\mathrm{F}\mathrm{M}{\left[0001\right]}_{2}^{A}$ to FM, though shifted to higher pressure for an increased tensile strain.

For *U*_eff_ ≥ 0.25 eV, see Fig. 6c, d, we find similar magnetic transitions as for ordinary PBE, but shifted towards more compressive strain and higher pressure. $\mathrm{A}\mathrm{F}\mathrm{M}{\left[0001\right]}_{2}^{A}$ is the favoured spin configuration at low pressure. Focusing on *U*_eff_ = 0.25 eV at *a*_0_ = 2.90 Å (0%), we find that at 9.1 GPa there is a transition from $\mathrm{A}\mathrm{F}\mathrm{M}{\left[0001\right]}_{2}^{A}$ to FM associated with a significant change in both magnetic moment and crystal structure, shown in Fig. 5b, c, as compared to a $\mathrm{A}\mathrm{F}\mathrm{M}{\left[0001\right]}_{4}^{A}$ to FM transition.

These results thus indicate that through manipulation of the strain within the thin film plane, e.g., through the choice of substrate, it is possible to strain-engineer accessible spin configurations. Moreover, through exertion of an external force, e.g., by applying a rather moderate pressure perpendicular to the film plane, one would enable a magnetic transition from AFM to FM. The significant change both in terms of crystal and magnetic structure during this transition should allow experimental verification.

### Magnetocrystalline anisotropy energy

There are so far no studies reported on the magnetocrystalline anisotropic energy (MAE) for Mn_2_GaC, neither experimental nor theoretical. Measurements on a related phase where 50% Mn in Mn_2_GaC is substituted for Mo in (Mo_0.5_Mn_0.5_)_2_GaC shows that the easy axis is in-plane, despite having a small MAE of 4.5 ± 0.3 kJ/m^3^ at 100 K favoring an out-of-plane orientation ^21^. Vibrating sampling magnetometry (VSM) measurements on Mn_2_GaC demonstrates that spins are aligned within the Mn planes^26,27^. We chose to investigate the MAE for Mn_2_GaC by including the spin–orbit coupling and by using the PBE + U exchange–correlation functional for 0 ≤ *U*_eff_ ≤ 1 eV. Based on results presented in previous sections of this work and in related experimental results^26–28^, we only consider the three low-energy collinear spin configurations, i.e., FM, $\mathrm{A}\mathrm{F}\mathrm{M}{\left[0001\right]}_{2}^{A}$, and $\mathrm{A}\mathrm{F}\mathrm{M}{\left[0001\right]}_{4}^{A}$.

**Figure 7:**
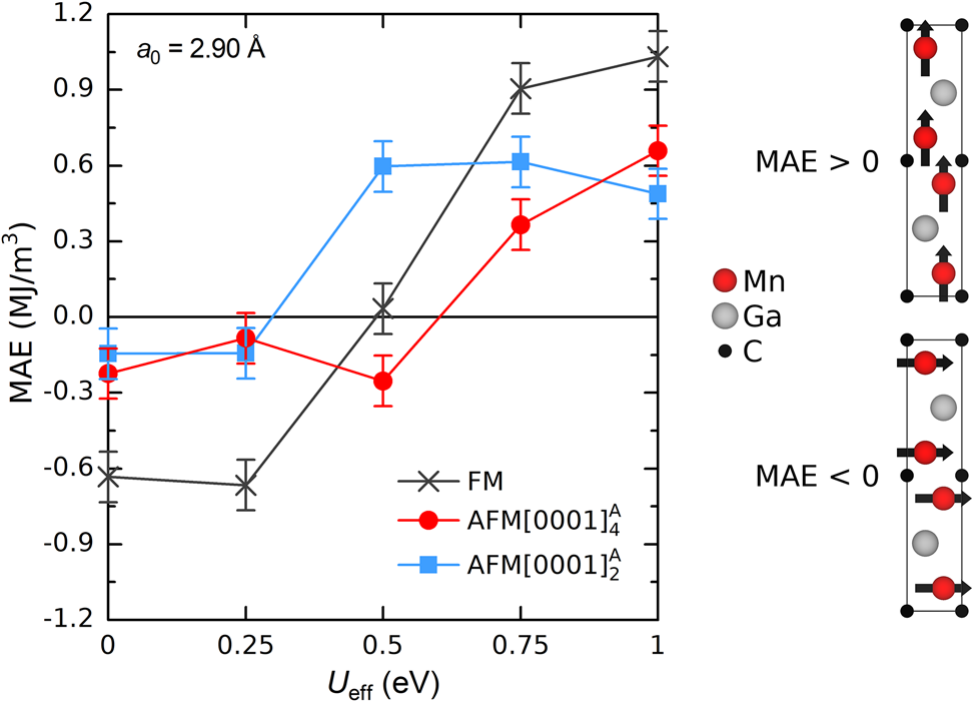
Magnetic anisotropy energy (MAE) as a function *U*_eff_ when considering FM, $\mathrm{A}\mathrm{F}\mathrm{M}{\left[0001\right]}_{4}^{A}$, and $\mathrm{A}\mathrm{F}\mathrm{M}{\left[0001\right]}_{2}^{A}$ spin configurations and using the PBE + U exchange–correlation functional. The estimated error bar from k-point convergence is 0.1 MJ/m^3^. The schematic shows the preferential spin alignment dependent on sign of the MAE.

The MAE in Fig. 7 was calculated as the energy difference between the configurations with spins aligned in-plane, i.e. parallel to the Mn layer, and normal to the Mn layers, MAE = E( →) − E(↑). To ensure convergence for calculated MAE we considered various k-point densities, see Fig. S11. From this we estimated an error of ± 0.1 MJ/m^3^. The structures used are for a_0_ = 2.90 Å and for each *U*_eff_ we used an optimized structure with c given Fig. 5b.

For *U*_eff_ ≤ 0.25 eV, we find negative values for MAE which is indicative of preferential spin alignment within the Mn planes. Note that this is consistent with experimental reports^26,27^. At larger *U*_eff_, MAE change sign and for *U*_eff_ ≥ 0.75 eV all spin configurations show preferential for out-of-plane spin alignment. Here we again notice how sensitive the results are with respect to value of *U*_eff_ and that results for *U*_eff_ > 0.25 are inconsistent with experiments. In addition, for ordinary PBE, we find a negative MAE for various *a* and *c* values, see Fig. S12, showing that FM and $\mathrm{A}\mathrm{F}\mathrm{M}{\left[0001\right]}_{4}^{A}$ having spins aligned in-plane become energetically more favorable upon tensile in-plane and compressed out-of-plane strain. $\mathrm{A}\mathrm{F}\mathrm{M}{\left[0001\right]}_{2}^{A}$, on the other hand, show a MAE value being almost constant for here considered lattice parameters.

Our results of having preferential spin alignment within the Mn planes, for *U*_eff_ ≤ 0.25 eV, are consistent with VSM measurements on Mn_2_GaC showing that spins are aligned within the Mn planes^26,27^. For $\mathrm{A}\mathrm{F}\mathrm{M}{\left[0001\right]}_{4}^{A}$ and $\mathrm{A}\mathrm{F}\mathrm{M}{\left[0001\right]}_{2}^{A}$ the calculated MAE for *U*_eff_ ≤ 0.25 eV is in the range − 100 to − 300 kJ/m^3^. We suggest that the MAE for a Mn_2_GaC thin film could be increased by expanding the in-plane lattice parameter *a* and thus reducing the value of *c* to minimize the stress in the *c* direction, as exemplified in Fig. S12(c).

## Conclusion

We here present a first principles study of the structural and magnetic properties of the atomically laminated Mn_2_GaC, with emphasis on effect of biaxial in-plane strain, pressure, and choice of different exchange–correlation functionals, aiming towards a relevant description of the structural and magnetic properties attainable for Mn_2_GaC in thin film form. Here we note that previous theoretical results were primarily relevant for bulk synthesis conditions, i.e. for an equilibrium volume and energy. It is found that use of the PBE functional or PBE + U (*U*_eff_ ≤ 0.25 eV) gives both structural and magnetic properties in good quantitative agreement with available experimental data. PW91, PBEsol, AM05, and LDA functionals and/or use of an + U approach result in structures and magnetic characteristics not compatible with the same experimental reports. Through a magnetic ground-state search we find several noncollinear magnetic configurations which bears structural and magnetic resemblance to low-energy collinear candidates. Notable is that through strain-engineering by choice of substrate or applied pressure, different spin configurations may be accessible, which in turn suggest a tuning potential of the magnetic properties, including attaining a FM spin state. Furthermore, we also suggest that the easy axis in Mn_2_GaC is parallel to the atomic planes and that the MAE can be increased through strain engineering by expanding the in-plane lattice parameter *a*, thus reducing the value of the out-of-plane lattice parameter *c*, which strengthen the spin orientation parallel to the atomic planes. Finally, the results presented here suggest that a quantitative description of the structural and magnetic properties of Mn_2_GaC is possible using PBE, which opens the way for further computational studies of these and related materials.

### Computational details

All first-principles density functional theory (DFT) calculations are performed using the projector augmented wave method^44,45^ as implemented within the Vienna ab initio simulation package (VASP)^46–48^, with a plane-wave energy cutoff of 400 eV. For sampling of the Brillouin zone we used the Monkhorst–Pack scheme^49^. Within this work we use different exchange–correlation functionals to study their dependence on structure and magnetic properties. If not stated otherwise, the generalized gradient approximation (GGA) as parameterized by Perdew-Burke-Ernzerhof (PBE)^50,51^ has been used. In parts of the work we also used the PBE revised for solids (PBEsol)^52^, the Perdew–Wang 91 (PW91)^53^, and AM05^54–56^ GGAs, and the local density approximation (LDA)^57^ functionals. In addition, we also used the rotationally invariant approach as proposed by Dudarev^41^. Note that within this formalism the onsite Coulomb parameter *U* and the exchange parameter *J* are spherically averaged into a single effective interaction parameter *U*_eff_ = *U* − *J* which does not depend on their individual values. The equilibrium structures are obtained by minimization of the total energy for an *a* and *c* lattice parameter grid with full relaxation of atomic positions until forces are converged below 10^–4^ eV Å^−1^. Calculations including spin–orbit coupling (SOC) have been performed in the mode implemented in VASP by Hobbs et al.^58^ and Marsman and Hafner^59^, in a two-step procedure. First, we performed a scalar-relativistic calculation to obtain the correct geometry, followed by calculations including spin–orbit coupling with spins aligned along the *c*-axis, i.e. parallel/antiparallel to [0001] axis, and perpendicular to the *c*-axis, i.e. parallel/antiparallel to both [1–100] and [1–210] axis.

**Figure 8:**
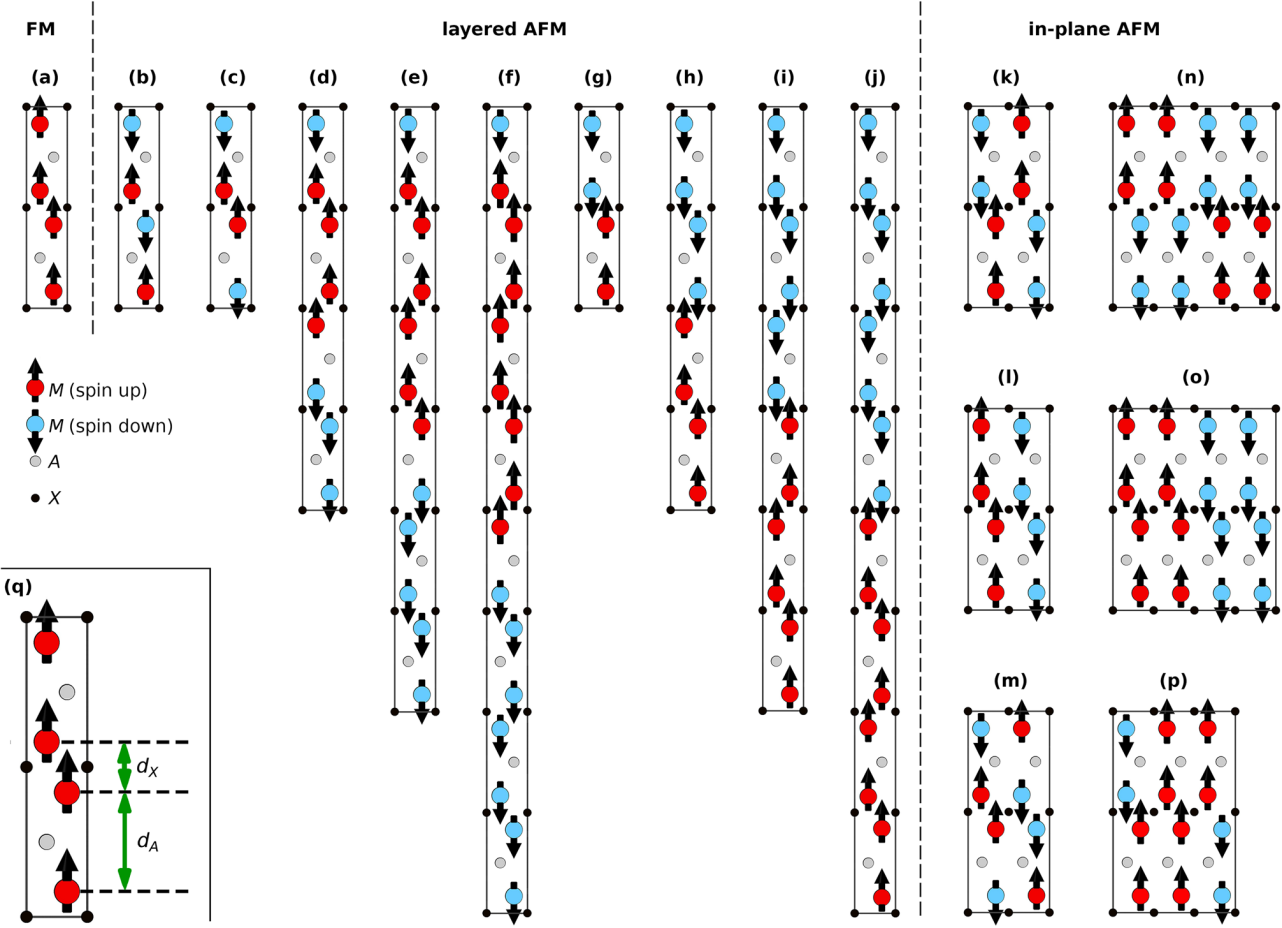
Schematic representation of collinear spin configurations for a *M*_2_*AX* phase where **(a)** represents ${\text{FM}}$, **(b)** $\mathrm{A}\mathrm{F}\mathrm{M}{\left[0001\right]}_{1}$, **(c)** ${\text{AFM}}{\left[0001\right]}_{2}^{A}$, **(d)** ${\text{AFM}}{\left[0001\right]}_{4}^{A}$, **(e)** ${\text{AFM}}{\left[0001\right]}_{6}^{A}$, **(f)** ${\text{AFM}}{\left[0001\right]}_{8}^{A}$, **(g)** ${\text{AFM}}{\left[0001\right]}_{2}^{X}$, **(h)** ${\text{AFM}}{\left[0001\right]}_{4}^{X}$, **(i)** ${\text{AFM}}{\left[0001\right]}_{6}^{X}$, **(j)** ${\text{AFM}}{\left[0001\right]}_{8}^{X}$, **(k)** in-${\text{AFM}}1$, **(l)** in-${\text{AFM2}}$, **(m)** in-${\text{AFM3}}$, **(n)** in-${\text{AFM4}}$, **(o)** in-${\text{AFM5}}$, and **(p)** in-${\text{AFM6}}$. **(q)** The interlayer distances *d*_*X*_ and *d*_*A*_ is indicated. Projections are viewed along the $\left[1-210\right]$ direction.

We have considered several collinear magnetic spin configurations for Mn_2_GaC, most of them have been defined previously for a general *M*_2_*AX* phase^27,38,39^. A schematic representation is shown in Fig. 8, and includes a ferromagnetic (FM), nine layered antiferromagnetic (AFM), and six in-plane AFM spin configurations. The notation used for layered AFM spin configurations is defined as follows; single layer AFM with spins changing sign for every *M* layer corresponds to AFM[0001]_1_, and multilayered AFM ordering with α consecutive *M* layers (where α = 2, 4, 6, 8) of the same spin direction before changing sign upon crossing an *A* or an *X* layer corresponds to ${\text{AFM}}{\left[0001\right]}_{\alpha }^{A}$ and ${\text{AFM}}{\left[0001\right]}_{\alpha }^{X}$, respectively^27,38^. In addition, the paramagnetic (PM) have been modelled using the disorder local moment (DLM)^60^ approach where the spin-correlation functions are equal to zero for at least the first 10 M-coordination shells. The disordered magnetic moments in (Mn↑_0.5_Mn↓_0.5_)_2_GaC is simulated by means of the special quasi-random structure (SQS) method^61,62^ using a supercell with 64 Mn, 32 Ga, and 32 C atoms, i.e., 4 × 4 × 1 or 16 *M*_2_*AX* unit cells. A strict definition of these collinear spin configurations is given in Table S1 where the spin correlation function Φ_*i*_ is given for the first 10 M-shells.

**Figure 9:**
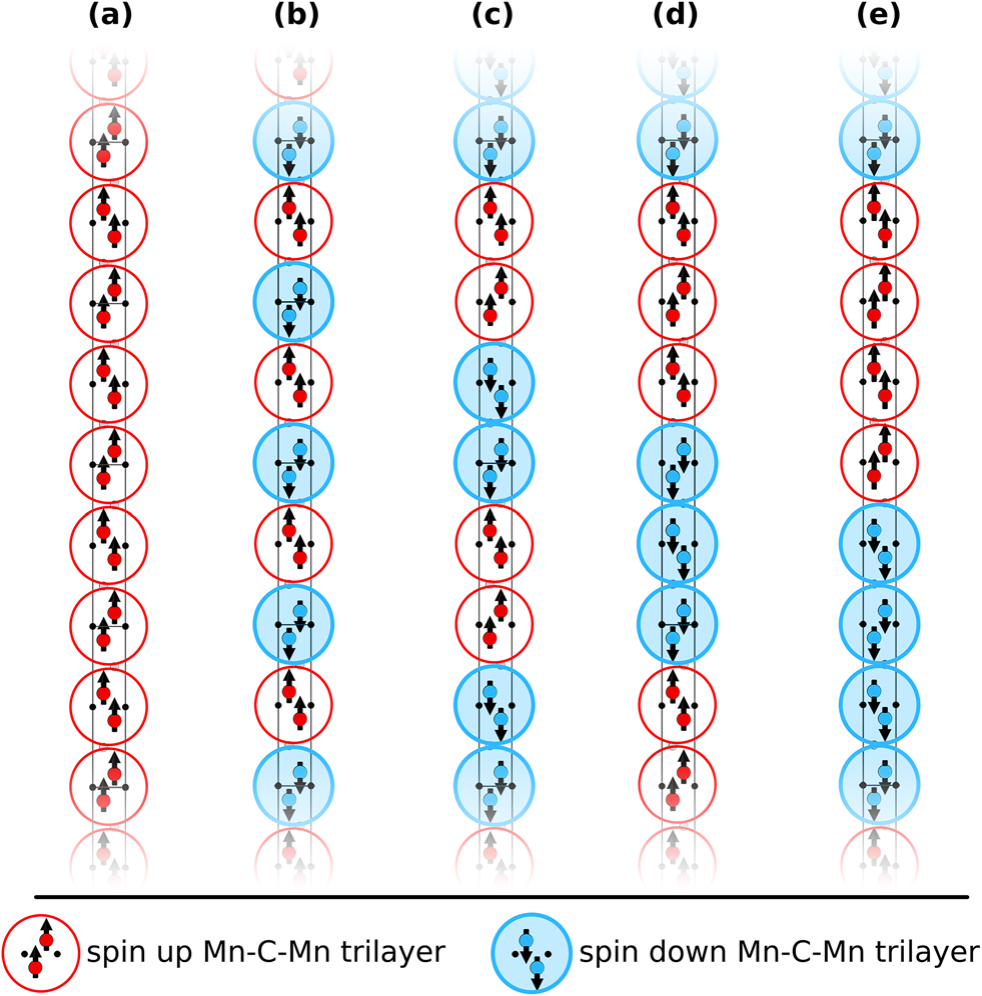
Schematic illustration of the coarse-grained super-moment model where each Mn-C-Mn trilayer is represented by a super-moment bead, red for spin up and blue for spin down, in Mn_2_GaC for **(a)** FM, **(b)** $\mathrm{A}\mathrm{F}\mathrm{M}{\left[0001\right]}_{2}^{A}$, **(c)** $\mathrm{A}\mathrm{F}\mathrm{M}{\left[0001\right]}_{4}^{A}$, **(d)** $\mathrm{A}\mathrm{F}\mathrm{M}{\left[0001\right]}_{6}^{A}$, and **(e)** $\mathrm{A}\mathrm{F}\mathrm{M}{\left[0001\right]}_{8}^{A}$ spin configurations.

For the magnetic ground-state search we used a coarse-grained model, shown in Fig. 9 in Computational Details, where the local moment of Mn atoms in a Mn-C-Mn trilayer plane is represented by a supermoment. This is motivated by all low-energy spin configurations of Mn_2_GaC having parallel spin directions within their Mn-C-Mn trilayers. The supermoment model is thus described using only magnetic exchange interactions (MEI) across the *A* layer along the *c* axis and cannot be used to modelling critical temperature since the MEI within a Mn-C-Mn tri-layer is neglected, and hence the exact temperature from the Monte Carlo simulation becomes irrelevant as it scales with the area of each layer. The spin correlation function Φ_*i*_ for the five considered spin configurations are given in Table S2. The essence of the approach is to identify possible non-trivial spin configurations through us of Monte Carlo simulations using a Heisenberg Hamiltonian.

2 $$\mathcal{H}=-\sum (i\ne j){J}_{ij}{\mathbf{e}}_{{\varvec{i}}}\cdot {\mathbf{e}}_{{\varvec{j}}},$$

where ${J}_{ij}$ is the MEI between pairs of super-moments (*i*, *j*), with unit vectors **e**_*i*_ and **e**_*j*_ along the local magnetic moment at site *i* and *j,* represented by a chain of super-moments. The exchange interactions *J*_ij_’s are derived for the first four super-moment interlayer coordination shells using the magnetic Connolly-Williams structure inversion method^42,43^, shown in Table S2 in combination with energies from first-principles calculations. To avoid possible metastable solutions, we initially set the temperature to a large value, and then slow cooling towards 0 K and to allow long-range magnetic interactions we used supermoment chains including up to 40 beads.

## Supplementary information

Supplementary Information.
