# Supplementary material for: Impact of strain, pressure, and electron correlation on magnetism and crystal structure of Mn2GaC from first-principles
Source: Sci Rep. 2020 Jul 9;10:11384. doi: 10.1038/s41598-020-68377-5 (PMC7347948; doi:10.1038/s41598-020-68377-5)
Supplement: Supplementary file 1 — Supplementary Information. [file 41598_2020_68377_MOESM1_ESM.pdf]

Supplementary information

Impact of strain, pressure, and electron correlation on magnetism and crystal structure of Mn2GaC from first-principles

Martin Dahlqvist and Johanna Rosen

Thin Film Physics, Department of Physics, Chemistry and Biology (IFM), Linköping University, SE-581 83 Linköping, Sweden

TABLE S1. Spin correlation functions Φ_i for the 10 first *M*-shells for FM and 15 AFM spin configurations as well as DLM for a M_2AX phase. n_α represents the number of *M* atoms in shell α.

Spin configuration	Spin correlation function for the 10 first *M*-shells										M_2AX unit cells
	Φ_1	Φ_2	Φ_3	Φ_4	Φ_5	Φ_6	Φ_7	Φ_8	Φ_9	Φ_{10}	
FM	1	1	1	1	1	1	1	1	1	1	1× 1 ×1
$AFM[0001]_1$	-1	1	-1	-1	-1	1	-1	1	-1	1	1× 1 ×1
$AFM[0001]_2^A$	1	1	1	-1	1	1	-1	1	1	-1	1× 1 ×1
$AFM[0001]_4^A$	1	1	1	0	1	1	0	1	1	0	1× 1 ×2
$AFM[0001]_6^A$	1	1	1	1/3	1	1	1/3	1	1	1/3	1× 1 ×3
$AFM[0001]_8^A$	1	1	1	1/2	1	1	1/2	1	1	1/4	1× 1 ×4
$AFM[0001]_2^X$	-1	1	-1	1	-1	1	1	1	-1	-1	1× 1 ×1
$AFM[0001]_4^X$	0	1	0	1	0	1	1	1	0	0	1× 1 ×2
$AFM[0001]_6^X$	1/3	1	1/3	1	1/3	1	1	1	1/3	1/3	1× 1 ×3
$AFM[0001]_8^X$	1/2	1	1/2	1	1/2	1	1	1	1/2	1/2	1× 1 ×4
in-AFM1	-1/3	-1/3	1	1	-1/3	-1/3	-1/3	1	-1/3	-1/3	1× 2 ×1
in-AFM2	1/3	-1/3	-1	1	1/3	-1/3	-1/3	1	1/3	1/3	1× 2 ×1
in-AFM3	2/3	1/3	0	1	0	-1/3	1/3	-1/3	-2/3	2/3	1× 2 ×1
in-AFM4	1/3	-1/3	-1	-1	1/3	-1/3	1/3	1	1/3	-1/3	1× 4 ×1
in-AFM5	1/9	1/9	5/9	1	-1/3	-1/3	1/9	1/9	5/9	1/9	1× 4 ×1
in-AFM6	-2/3	1/3	0	1	0	-1/3	1/3	-1/3	2/3	- 2/3	1× 3 ×1
DLM	0	0	0	0	0	0	0	0	0	0	4× 4 ×1
Definition of *M*-shell	1st across *X*-layer	1st in *M*-plane	2nd across *X*-layer	1st across *A*-layer	3rd across *X*-layer	2nd in *M*-plane	2nd across *A*-layer	2nd in *M*-plane	4th across *X*-layer	1st across *A*- and *X*-layer	
n_α:	3	6	3	1	6	6	6	6	6	6	

TABLE S2. Spin correlation functions Φ_i for the first four coordination shells of the five coarse-grained magnetic spin configurations within the coarse-grained super-moment model used in Heisenberg Monte Carlo simulations. n_α represents the number of super-moments in shell α.

Spin configuration	Spin correlation function			
	Φ_1	Φ_2	Φ_3	Φ_4
FM	1	1	1	1
$AFM[0001]_2^A$	-1	1	-1	1
$AFM[0001]_4^A$	0	-1	0	-1
$AFM[0001]_6^A$	1/3	-1/3	-1	-1/3
$AFM[0001]_8^A$	1/2	0	-1/2	-1
n_α:	2	2	2	2

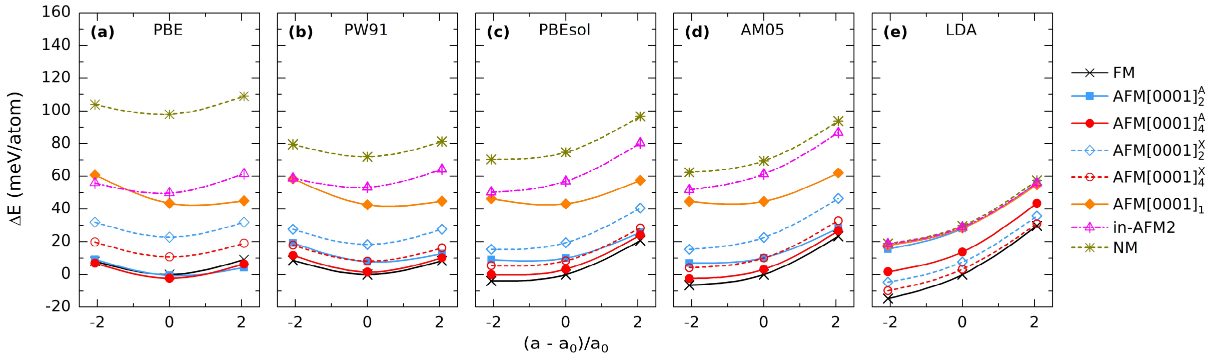

Figure S1. Energy relative ferromagnetic solution with $a_0 = 2.90$ Å, ΔE, as function of strain for FM, $\mathrm{AFM}[0001]_2^A$, $\mathrm{AFM}[0001]_4^A$, $\mathrm{AFM}[0001]_1$, $\mathrm{AFM}[0001]_2^X$, $\mathrm{AFM}[0001]_4^X$, in-AFM2, and NM spin configurations using (a) PBE, (b) PW91, (c) PBEsol. (d) AM05, and (e) LDA exchange-correlation functionals.

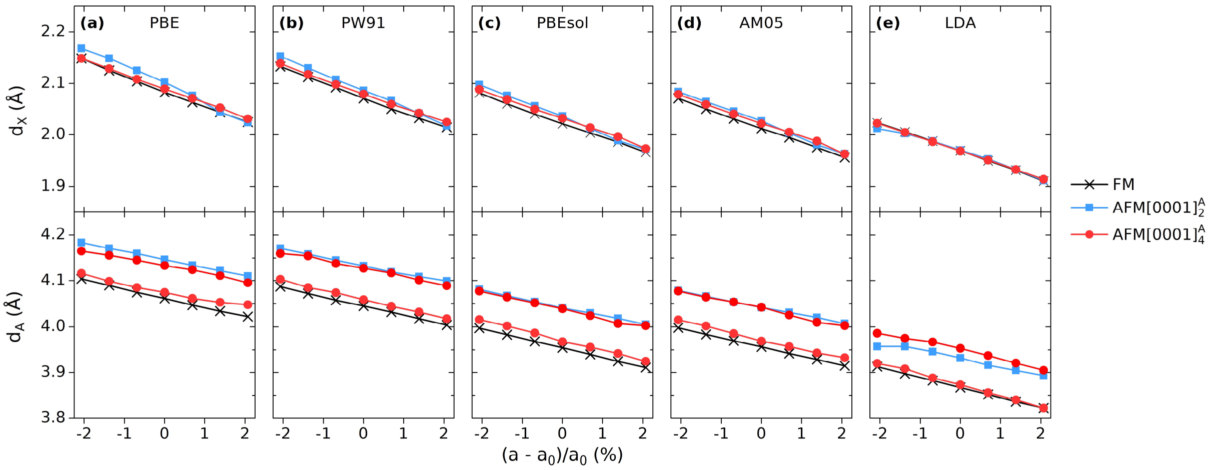

Figure S2. Interlayer distance d_X (top panels) and d_A (bottom panels) as function of biaxial in-plane strain for FM, $\mathrm{AFM}[0001]_2^A$, and $\mathrm{AFM}[0001]_4^A$ spin configurations using (a) PBE, (b) PW91, (c) PBEsol, (d) AM05, and (e) LDA exchange-correlation functionals

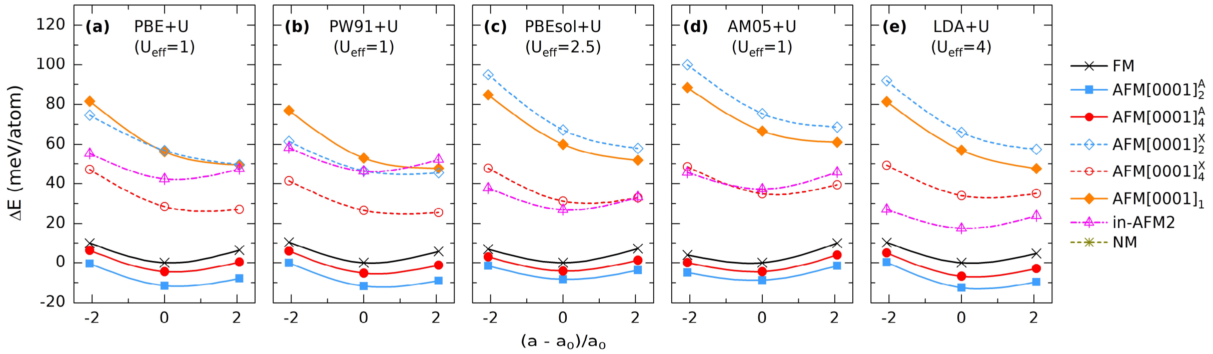

Figure S3. Energy relative ferromagnetic solution with $a_0 = 2.90$ Å, ΔE, as function of strain for FM, $\mathrm{AFM}[0001]_2^A$, $\mathrm{AFM}[0001]_4^A$, $\mathrm{AFM}[0001]_1$, $\mathrm{AFM}[0001]_2^X$, $\mathrm{AFM}[0001]_4^X$, in-AFM2 and NM (not within range) spin configurations using (a) PBE+U, (b) PW91+U, (c) PBEsol+U, (d) AM05+U. and (e) LDA+U exchange-correlation functionals. Considered U_{eff} values are given within each panel.

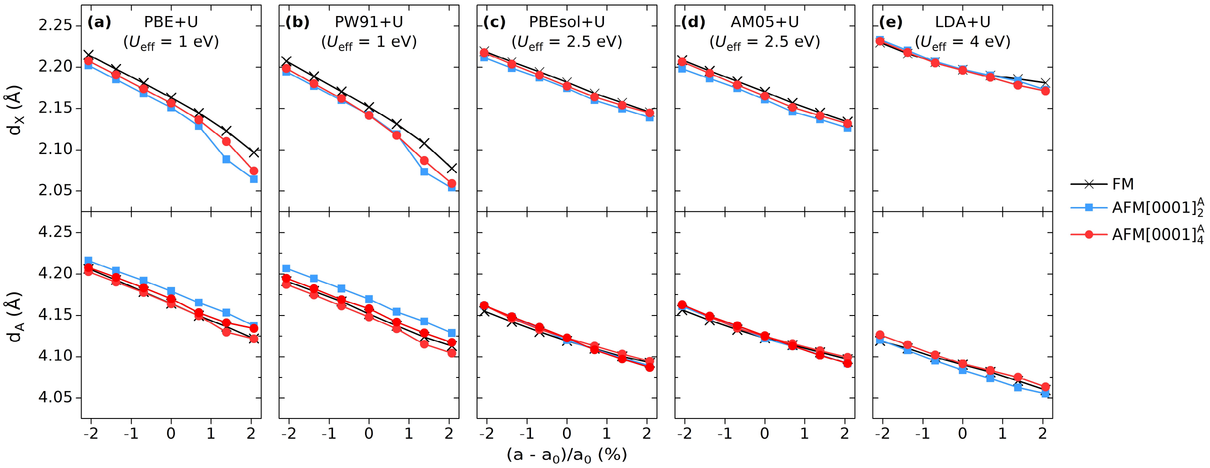

Figure S4. Interlayer distance d_X (top panels) and d_A (bottom panels) as function of biaxial in-plane strain for FM, AFM$[0001]_2^A$, and AFM$[0001]_4^A$ spin configurations using (a) PBE+U, (b) PW91+U, (c) PBEsol+U, (d) AM05+U. and (e) LDA+U exchange-correlation functionals. Considered U_{eff} values are given within each panel.

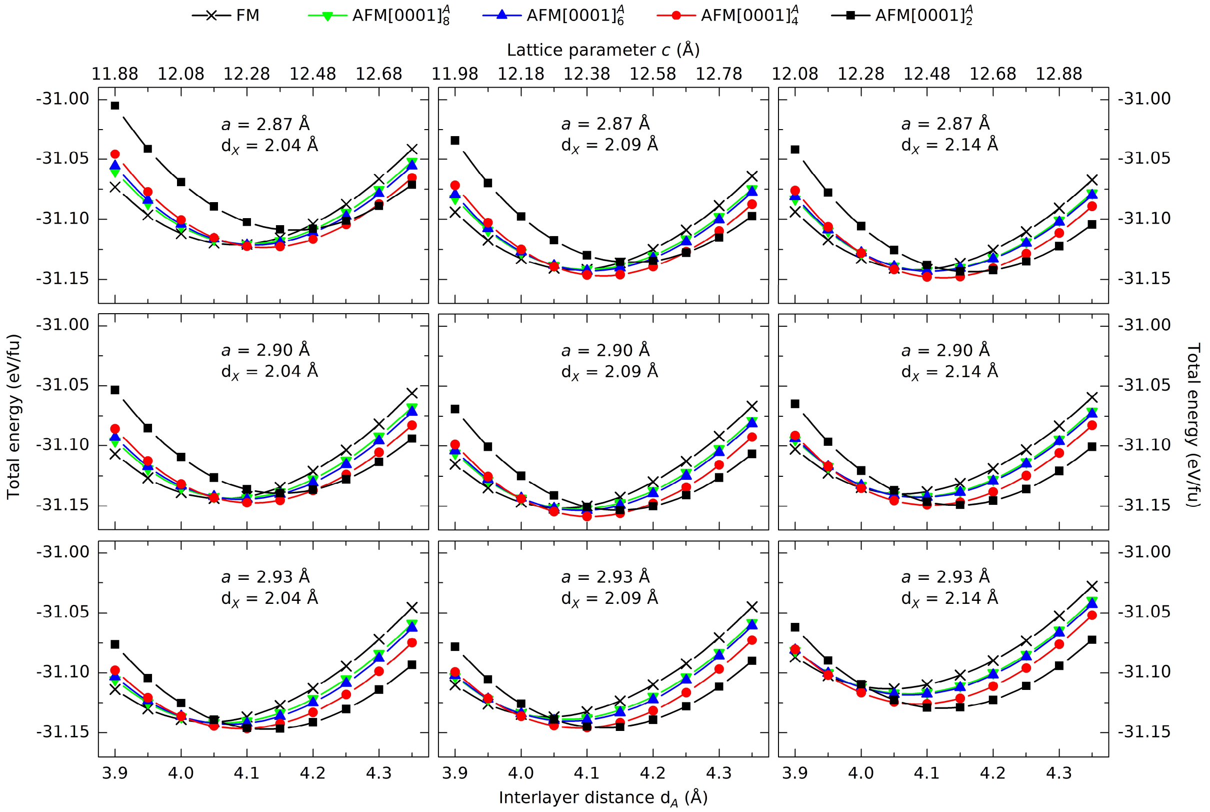

Figure S5. Energy as a function of interlayer distance d_A for nine series of different lattice parameter a and interlayer distance d_X.

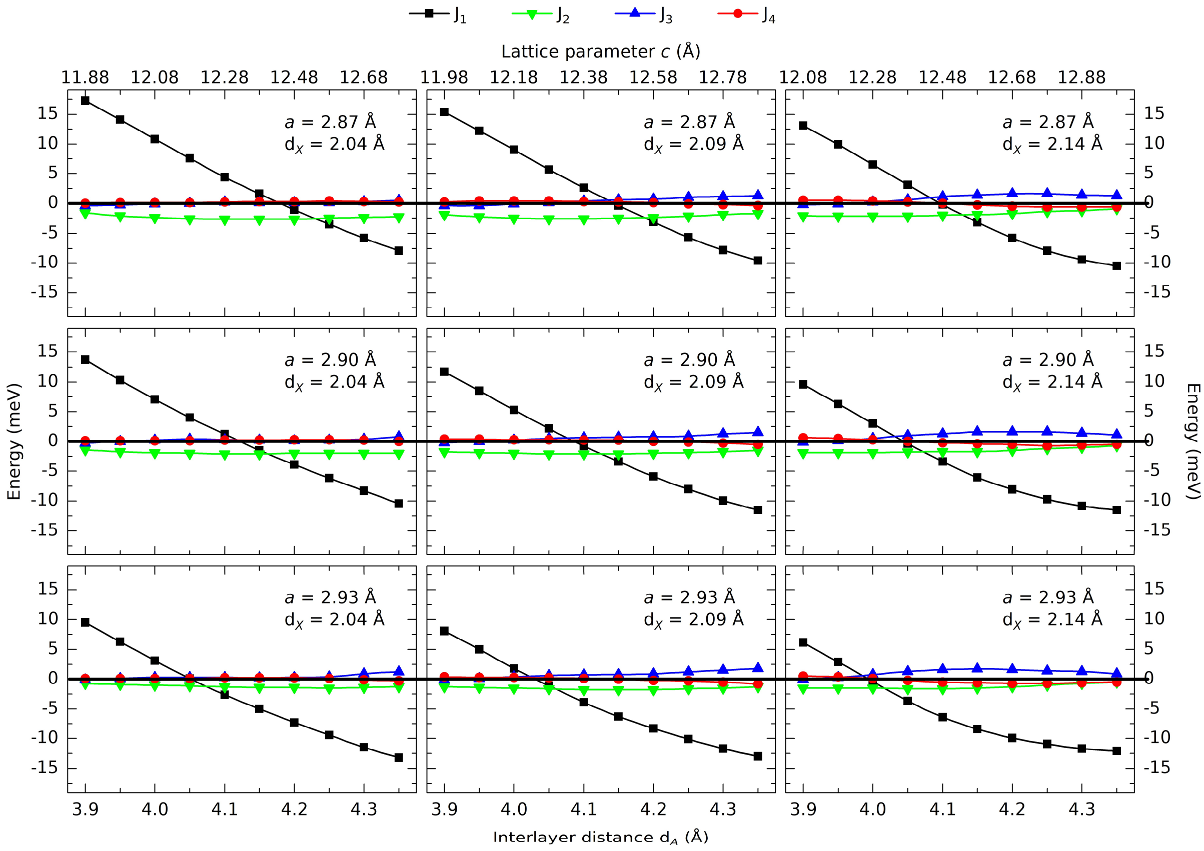

Figure S6. Exchange interaction parameters J_{ij} for the four nearest neighbouring shells as a function of interlayer distance d_A for nine series of different lattice parameter a and interlayer distance d_X. Generated from energies given in Figure S5.

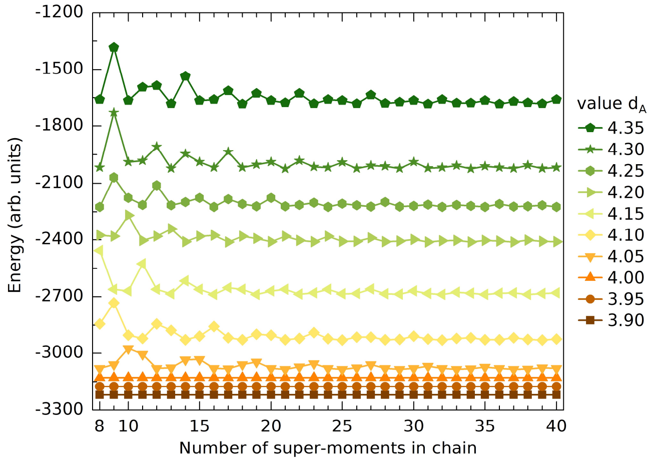

Figure S7. Total energy from Heisenberg Monte Carlo simulations as function of number of supermoment beads for various interlayer distances d_A. For this set of simulations we used input from first-principles calculations where $a = 2.90$ Å and $d_X = 2.09$ Å.

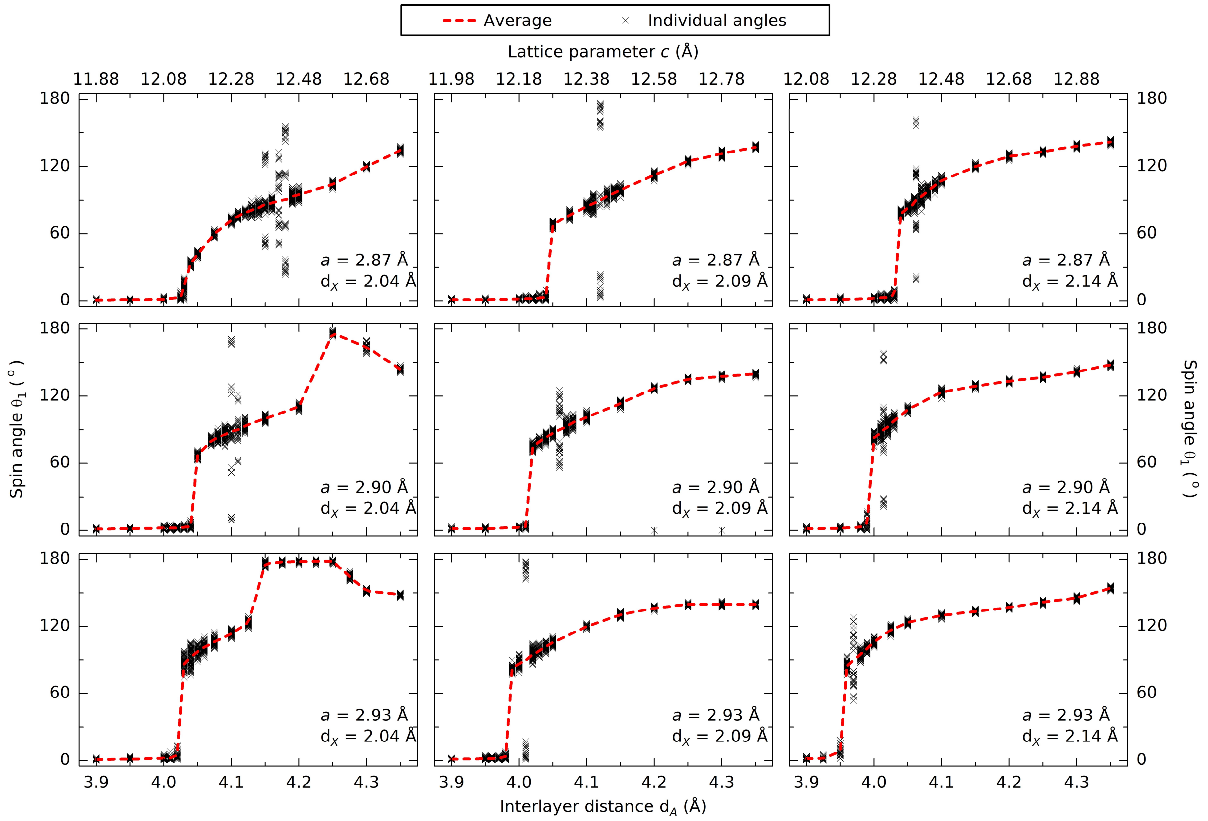

Figure S8. Angle between nearest neighbor super moment spin vector θ_1 as a function of interlayer distance d_A for nine series of different lattice parameter a and interlayer distance d_X when using exchange interaction parameters J_{ij} in Figure S6 as input for the Heisenberg Monte Carlo simulations.

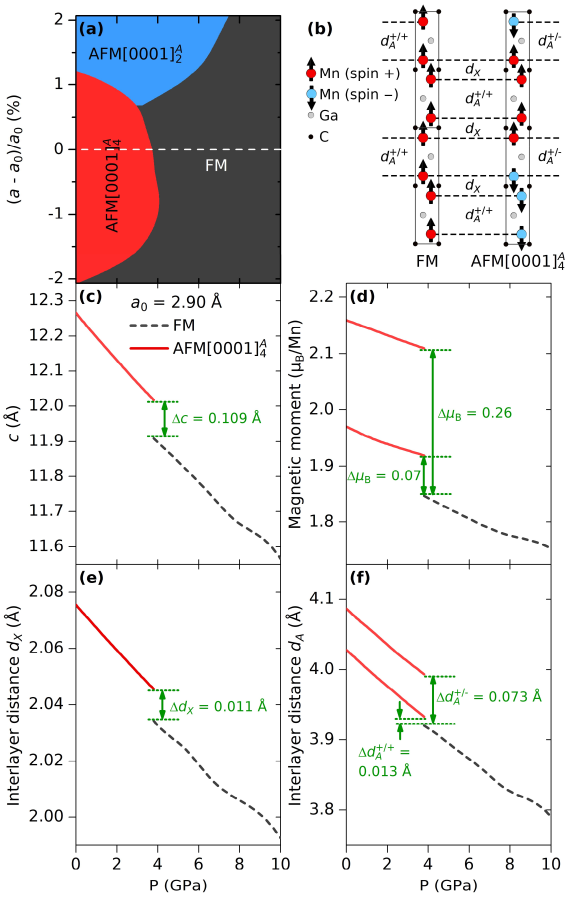

Figure S9. (a) Spin configuration of lowest enthalpy on biaxial-in-plane strain – pressure grid. The experimentally measured lattice parameter a of 2.90 Å is represented by the horizontal dashed line [23, 24] (b) Schematic illustration of FM and AFM$[0001]^A_4$ spin configurations where the interlayer distances d_X and d_A is indicated. (c) Lattice parameter c, (d) local magnetic moment μ_B, interlayer distance (e) d_X and (f) d_A as function of applied pressure for $a_0 = 2.90$ Å. At the transition from AFM$[0001]^A_4$ to FM, the absolute change in c, μ_B, d_X and d_A, is given. Results from calculations using the of PBE exchange correlation functional.

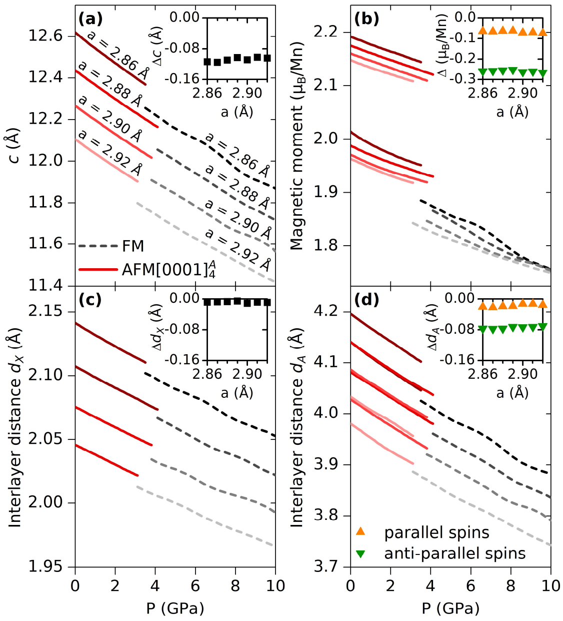

Figure S10. (a) Lattice parameter c, (b) local magnetic moment μ_B, interlayer distance (c) d_X and (d) d_A as function of applied pressure for four values of a. Red colours correspond to AFM$[0001]^A_4$ and black-grey to FM spin configurations. The insets in each panel show the absolute change as function of for the transition from AFM$[0001]^A_4$ to FM. Results from calculations using the of PBE exchange correlation functional.

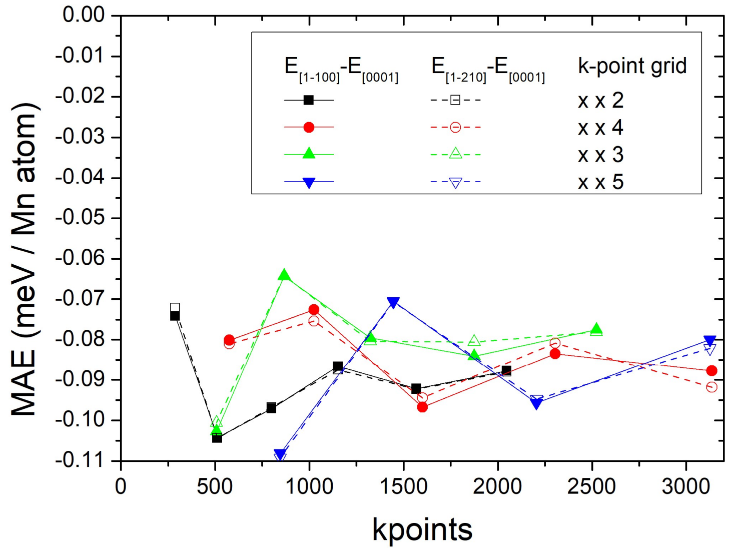

Figure S11. Example of energy difference convergence as function number of *k*-points for various *k*-point grids, FM spin configuration, and use of PBE exchange correlation functional.

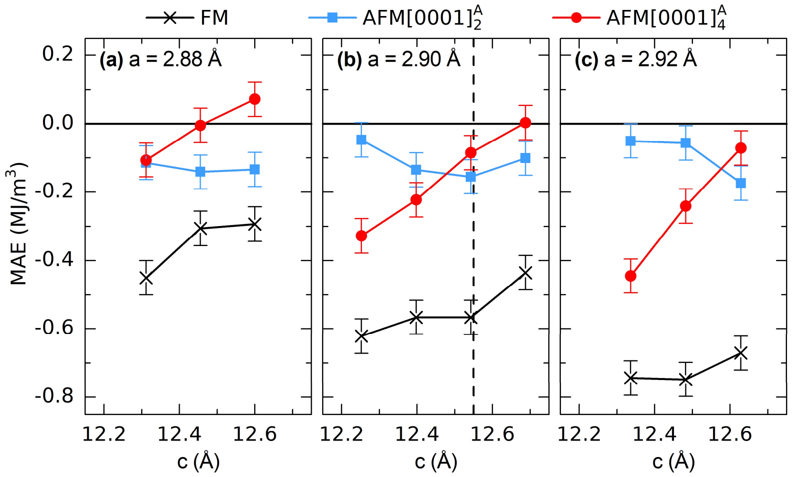

Figure S12. Magnetic anisotropy energy (MAE) as function of lattice parameter *c* where the in-plane lattice parameter *a* is (a) 2.88 Å, (b) 2.90 Å, and (c) 2.92 Å, when considering FM, AFM[0001]$_2^A$, and AFM[0001]$_4^A$ spin configurations and using the PBE exchange-correlation functional. The estimated error bar from k-point convergence is 0.1 MJ/m^3. The experimentally measured lattice parameter *c* of 12.55 Å is represented by the vertical dashed line in (b) [23, 24].
